# Computation of Fetal Kicking in Various Fetal Health Examinations: A Systematic Review

**DOI:** 10.3390/ijerph19074366

**Published:** 2022-04-05

**Authors:** Yuwei Liu, Rongrong Xuan, Yuhuan He, Feng Ren, Yaodong Gu

**Affiliations:** 1 Faculty of Sports Science, Ningbo University, Ningbo 315211, China; zoeliuyuwei@163.com (Y.L.); heyuhuan@nbu.edu.cn (Y.H.); guyaodong@nbu.edu.cn (Y.G.); 2 Affiliated Hospital of Medical School, Ningbo University, Ningbo 315020, China; 3 Department of Physical and Health Education, Udon Thani Rajabhat University, Udon Thani 41000, Thailand; 4 Faculty of Informatics, Eötvös Loránd University, H-9700 Szombathely, Hungary

**Keywords:** fetal movement, kicking, ultrasound, cine-MRI, biomechanical

## Abstract

Fetal movement has always been considered an essential indicator to evaluate the health of the unborn fetus. Many factors affect fetal movement. The frequency of fetal kicking is an important measurement of whether fetal development is progressing and healthy. Various instruments and methods of detecting fetal movement have been used and each method has its advantages and disadvantages. Although limited by the fetal environment in utero, the finite element method and musculoskeletal model can be used to calculate fetal lower limb movement. This review aims to summarize the current detection techniques for fetal movement, especially in the lower limbs. These will be outlined by describing the different measurements of fetal movement, and the related biomechanical analyses of fetal lower limb skeletogenesis and the associated muscular development to better evaluate and calculate the movements of the fetus in the womb.

## 1. Introduction

Obstetricians discovered the relationship between fetal movement reduction and stillbirth in the 1970s [1]. Sudden alterations in fetal activity include a sudden increase or decrease, or the disappearance of any fetal movement; these changes are often related to adverse fetal outcomes, especially when the fetus is about to be born. Fetal dysplasia or excessive fetal growth during the third trimester is most likely to result in reduced fetal movement. The outcomes include extremely low fetal weight, fetal growth restriction sometimes resulting in premature labor, and fetal death [2,3,4]. Although examination and measurement of the movement of the fetus prior to birth does not cause any additional physical burden on the pregnant mother or the fetus itself, there is still no specific protocol, such as surgery or drug therapy, to solve the anomalous variations in quickening [5,6]. Extremely vigorous motion in the late gestational phase may indicate that the risk of stillbirth increases threefold; nevertheless, this type of strenuous activity is usually determined in retrospective investigations [5,7]. It has been observed that there is a relationship between unusually vigorous movements and stillbirth [4].

Alterations in fetal motion may be pathological or physiological. Physiologically, there are many causes of fetal movement restriction. As the fetus grows, fetal growth tends to be limited by the uterine environment in the third trimester, which leads to decreased fetal movement. Pathologically, if oxygen and nutrients in the placenta are inadequate, this can lead to the condition of fetal hypokinesis [8,9]. Hypoxia and nutrient consumption cause the brain and central nervous system to save energy physiologically, and as a result, the frequency and intensity of fetal movement decreases. Maternal diseases and chromosomal abnormalities also cause reduced or absent fetal movement [10]. In addition to this, some pathological causes, such as placental dysfunction syndrome, may also result in fetal movement restriction, which ultimately leads to decompensation, and even stillbirth of the fetus [5,6,8,11]. Occasionally, fetal epileptic seizures associated with increased fetal movement can be accompanied by a lack of oxygen, leading to heart failure and possible death [12]. The motor behaviors of neonates, such as posture and reflexes, positively correlate with neurologic impairment. Therefore, the detection of neuropathological conditions related to fetal movements and reactions in the early stage is very crucial [13,14,15].

The first involuntary fetal movement begins around 7 gestational weeks; independent voluntary movement, such as kicking, commences at 12 weeks as the neuromuscular unit continues to grow [16]. Distinct movement patterns, such as startle, hiccup, stretch, and head or eye movements usually occur at about 15 gestational weeks [17]. Maternal sensation of fetal motion normally occurs after 16 weeks [18]. From the 24th week until the 32nd week of pregnancy, the frequency of fetal movement tends to be steady until labor is about to begin. The evaluation of fetal lower extremities is crucial for the evaluation of neurological disorders and muscular and skeletal development. Therefore, the detection of movement during the period from the gestational age of 24 weeks to postpartum 16 weeks of prematurity plays an essential role in detecting fetal neurodevelopmental abnormalities [19]. The type of fetus movement may change as the pregnancy progresses; nonetheless, during this same period, the frequency does not decrease except for pathological reasons [20]. The regular movement of the fetus has been regarded as one of the indicators of fetal health and represents the normal function of the fetal central nervous system [21,22].

Research has demonstrated via the observation of the movement of a large number of fetuses that in the third trimester, the fetal general range of movement decreases with the progress of the pregnancy. As the fetus grows in size, its range of motion in the womb becomes more limited. The quality and quantity of fetal motion are affected by space constraints in the uterus and the uterine environment. This is particularly prominent as the amplitude of fetal motion decreases near the end of labor. By about 30 weeks of gestation, lower limb motion decreases significantly, which may be related to high flexion of the hip and knee joints. In addition, full-term infants have lower knee and ankle joint angles than term-equivalent age preemies, and are more prone to bending the lower limbs [23]. Although the mechanical stimulation mechanism is unknown, there is a causal link between fetal motion and joint morphology [24]. When neurological dysfunction appears in the early trimester of pregnancy, fetuses often have lower limb disarray and uncontrollable movements throughout the whole body [25]. Therefore, the evaluation of fetal movement is also a notable predictor of neuropathology disorders, not just cerebral palsy or hemiplegia [14,26]. The qualitative and quantitative assessment of general fetal movement is a compatible method for evaluating neurological disorders, diseases, and congenital malformations with growth constraints in the fetus.

Unnatural fetal movement has always been a concern and it is an influential index for evaluating the health of fetal development. A pathological reduction in fetal movement may lead to several diseases, such as dysplasia of the hip, fetal akinesia deformation sequence, and arthrogryposis. With the advent of objective measurement tools, maternal perception is no longer the primary method of assessing fetal movement. Assessment methods such as cardiotocography (CTG), the actograph, ultrasound, and cine-MRI have been used more recently.

These methods have proved to be effective methods to detect fetal movement in clinics. However, it difficult to quantitatively analyze fetal movements accurately using these measurements, especially during late gestation. Therefore, the accuracy and effectiveness of fetal motion assessment are especially important. The most common lower limb movement of the fetus, especially the kicking movement, is significant in the study of fetal maturation and movement. The biomechanical detection and measurement of fetal lower limb movement can provide more accurate data to support fetal whole-body movement and health evaluation. In this study, our aim was to conduct a systematic review to (i) summarize the existing measurement methods for assessing fetal kicking movement and (ii) determine to what extent each method can measure fetal lower limb movement.

## 2. Materials and Methods

**Figure 1:**
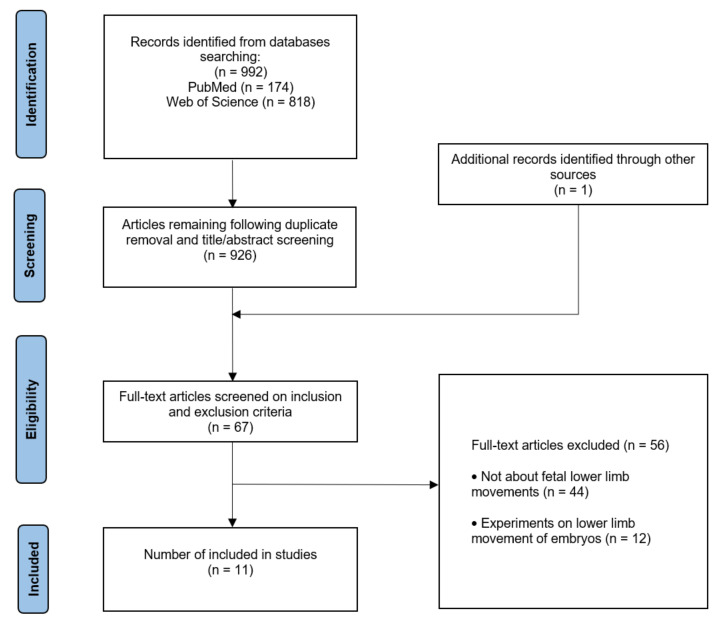
PRISMA flowchart of the process including the literature search and screening.

This systematic review followed PRISMA (Preferred Reporting Item for Systematic Reviews and Meta-analysis) protocols [27]. The articles chosen for this review were acquired using searches on PubMed and the Web of Science up until March 2022. Outcomes of interest were conclusions about fetal movements or lower limbs. In the search strategy, the keywords consisted of fetal movement biomechanics and the terms using the Boolean operation keywords in this search were: (fetal OR foetus* OR embryo OR fetus OR foetal) AND (move* OR motion OR motility OR kick*) AND (“lower limb” OR “lower extremity” OR biomechanics* OR musculoskeletal). In total, 992 articles were found in the chosen databases and 11 were selected for this systematic review. Animal experiments and cadaver experiments did not meet the selection criteria of this paper and were excluded. Only English papers and journal papers are selected, and the paper screening process is shown in Figure 1. According to PICO principles (participants, interventions, comparisons, and outcomes), this study seeks the computation of fetal kicking in various fetal health examinations. Information was extracted from 11 selected studies based on the subjects’ characteristics and biomechanical gait parameters. Data were collected, including the gestational age, numbers of fetus, reference method, ultrasonic scanning plane/MRI section thickness, and inspection area.

## 3. Results

An electronic database search of 992 articles on fetal movement was conducted and an additional study was found from a bibliography. Of these, 926 articles were discarded based on titles, abstracts, and duplication. The remaining 67 articles were read in total, and 56 were discarded according to the screening criteria. Finally, a total of 11 articles were included. The details are shown in Table 1.

## 4. Discussion

A series of methods have been used previously to quantify fetal movements, such as cardiotocography, ultrasound, or MRI, but each method requires localization, and it is difficult to determine a gold standard. Each test has its advantages and disadvantages; previously popular methods, such as CTG, for detecting fetal movement have proven ineffective and not reliable. As technology advances, it is becoming easier to capture fetal movement patterns more accurately and distinguish fetal movement from maternal sensation. Through the modeling of skeletal muscle, there is the potential to develop specific simulations of the force of fetal kicking.

### 4.1. Maternal Sensation

Maternal sensation was the most common method used to assess fetal movement. Mothers are able to judge the health of their baby by perceiving its movements. Pregnant females sense the movement of the fetus through the uterine and associated abdominal wall muscles, and the mother easily recognizes a reduction in the fetus’ movement. Factors such as fetal weight, fetus placental position, and maternal psychology all affect the maternal perception of fetal movement [38]. On account of the different approaches to counting the frequency of fetal movements, it is difficult to assign appropriate standard for evaluating normal fetal movement in late gestation [39,40]. Reduced fetal movement frequency perceived by the mother is associated with an increased risk of fetal stillbirth [41]. The fetal movement perceived by the mother is particularly evident in the fetal trunk and fetal lower limbs [18]. The actual fetal movement frequency is generally influenced by factors such as the mother’s body position, maternal activity, anxiety neurosis and food intake [42,43,44]. The increased strength and frequency of fetal motion presumably reflects a general trend across the previous two weeks instead of a simple uncharacteristic warning signal [45]. Excessive fear from the expectant mother may be why the pregnant woman feels the fetal movement is extremely intense [7,46].

Several observational studies have demonstrated that any additional changes in fetal activity prior to delivery may affect neonatal outcomes. When seizure-like fetal hyperactivity occurs, a number of potential causes of injury need to be considered. Harmful stimuli, abnormal spasms, and infection also need to be considered and distinguished from fetal movement increases [7,46,47]. The hypokinetic movement repertoire of most fetuses is often accompanied by low velocity and a reduction in the frequency of activity. Conversely, if the fetus appears to produce a sudden rapid action, this often prompts the occurrence of epilepsy. In follow-up visits of women with stillborn infants, sudden changes in the exaggerated activity in infants are sometimes described as crazy motions, as distinguished from some of the general movements of early infants [7,40]. About a quarter of women had negative outcomes such as stillbirth or preterm childbirth when fetal movements decreased. Reduced perception of fetal movement can usually be seen as a warning of intrauterine death [2,5,48].

### 4.2. Maternal Wearable Devices

Maternal wearable devices can be independent of expensive laboratory equipment such as ultrasound, and can detect fetal movement over a more extended period instead of a short clinical measurement period [49]. Most accelerometer-based systems such as capacitive accelerometers and tri-axial accelerometers are not able to distinguish between fetal and maternal voluntary movements. Some study results show that startle movements and other forms of activity can be differentiated by acoustic sensor systems in association with accelerometers, effectively eliminating inconsistent noisy data resulting from maternal movements [50]. The acoustic measurement of fetal movement outcomes is not as accurate as ultraphonic scans or MRI.

### 4.3. Cardiotocography (CTG)

Cardiotocography (CTG) is a well-developed test for detecting fetal movement. This technique is potentially essential in that compensatory ultrasound is used to identify heart rate to predict fetal hypoxia [51]. It can be used externally and internally to observe and preserve the fetal heart rate and contraction or deviation of the mother’s uterus placement. The results of the CTG test can be used to describe four features of fetal movement: fetal heart rate variability (FHRV), baseline fetal heart rate, acceleration, and deceleration. Although the CTG can measure fetal movement, the noise affecting the recordings make it less accurate; when the CTG is inclusive of accelerations, the results are practically in accordance with real-time ultrasonic imaging [52]. Intrauterine epilepsy is difficult to distinguish from the normal movement of the fetus and can only be confirmed when using ultrasound and CTG to check out the epilepsy phenomena at delivery [53]. Although the clinical effect of fetal movement reduction is not accurate and is strongly associated with stillbirth, CTG as a routine clinical method is mostly (about 80–90%) used with ultrasound to detect fetal motion reduction [54]. Recent research suggests that using CTG as a routine procedure for detecting fetal movement does not provide great advantage for diagnosis [55].

### 4.4. Actograph

The actograph is an objective method to quantify the data from CTG, and is positively associated with antenatal ultrasound assessment [56]. It can be used to distinguish high-frequency signals such as fetal heart activity and regards low-frequency signals as fetus movement [57]. Although the subtle movements of the fetus, such as breathing are not enough to produce any signals, the vast majority of fetal movement signals can be detected by the actograph [58]. The size of the fetus has a positive correlation with fetal movement using quantified actograph output. Actograph exhibits a high false-positive rate when it is included in most CTG devices, so it is not widely used [56].

### 4.5. Ultrasound

Two-dimensional sonography images were first used in the 1970s to observe the intrauterine environment of the fetus for real-time imaging [59]. Most maternal perceptions of fetal movement are usually so subjective that it is easily confused; only a few motions can be captured by the doppler device [60]. Modern brightness B-mode ultrasonography uses a curvilinear array of transducers to capture the three-dimensional image and video of the fetus in continuous motion. During an ultrasound examination, the obstetrician is required to apply the conductive gel to the appropriate examination area and record the relevant features of the fetal conditions. In order to minimize the exposure, the operator needs to monitor fetal movements for a brief period. Nevertheless, measurement of the fetal movement is generally not part of a routine prenatal ultrasonic examination. The variable speed, amplitude, direction of fetuses’ general movement disorders can be detected and analyzed by using sonographic imaging; this is an important tool for obstetricians to judge whether the fetus is healthy or not [61].

The volume of fetal growth fluid in the uterus detected by ultrasound to recognize placental insufficiency is closely related to fetal movement restriction and stillbirth [62]. Sonography is an optimum method to measure fetal movement. The only limitation to this approach isa segmentary view of the fetus’ gross movement around first 20 weeks [63]. In late gestation, Doppler ultrasound is not able to capture the whole fetus for clinicopathological examination due to the increase in fetal volume; natimortality of the fetus is still an intractable problem [57]. Therefore, the accuracy of fetuses’ motor evaluation in the later stages of pregnancy is hard to guarantee [64]. Ultrasound is still regarded as the best standard in fetal movement detection and is frequently used to produce quite clear two-dimensional images. The fetus’ general movement is usually suitable for ultrasound measurement, and is comprised of spontaneous motor behavior that is stable and sustainable from the first trimester of pregnancy and is maintained three to five months after delivery [61]. It was found that with the increase in gestational age, the frequency of all movement patterns decreased, which may be related to the uterine volume [65]. Although the measurement of fetal movements is momentous in fetal neurological measurements, with the restriction of using ultrasound during the second and third trimesters, it is not able to cover the whole movement using the standard method to measure the whole fetal movement [66]. Using ultrasound to observe fetal movement and intervention has reduced the fetal mortality rate; thus, it can also be inferred that using ultrasound and intervention therapy can effectively reduce fetal mortality [67].

### 4.6. Cine-MRI

The development of dynamic cine-MRI sequences in the field of MRI has led to improved techniques for detecting fetal movement during recent years. Cine-MRI scans are a novel and valuable technique that can directly observe the entire fetal movement using MRI technology [68]. The rendered image of cine-MRI can precisely capture the fetus’ gross body movement patterns and monitor the fetus in detail, especially in the equivalent period of delivery of the fetus [64]. Cine-MRI can provide more comprehensive imageology information of the intrauterine environment. Through the use of cine-MRI, it was found that changes in fetal motor behavior were positively related to the uterine cavity volume during pregnancy [33,69]. Cine-MRI can be used to identify premature and neonatal outcomes by distinguishing regular and unusual images of fetal movement [64]. Cinematography is considered the most suitable imageology method for fetal motion since its balanced steady-state free alignment makes the image more detailed and precise [70]. The cine-MRI has a lower absorption rate, and a higher signal-to-noise ratio compared to other alternative imaging methods under the same conditions. Moreover, it can also clearly display fluid and tissue boundaries, rendering images with strong contrast characteristics [68,70]. At around 28 gestational weeks, the fetus begins to have limited movement in the womb; cine-MRI can more easily distinguish changes in fetal position, as well as some bending and stretching, than other measuring methods. The fetus may occupy approximately 90% uterine content at near term, which decreases the movement of the fetus because the volume of the fetus in the uterine cavity increases [66].

Inborn brain impairments increase the risk of neurodevelopmental abnormalities after birth [71]. Evaluation of the pattern and frequency of movement during pregnancy is an efficacious method for neurological screening of fetuses with congenital brain injury [71]. Biomechanical testing combined with the cine-MRI and musculoskeletal modeling methods attempt to characterize fetal movement quantitatively. Finite element analysis and musculoskeletal measurement can quantify the kick forces and associated muscle forces, as well as fetal bone stress and strains [35,72,73]. The lower limb muscle forces generated by kicking at a certain gestational age can be quantified using the finite element analysis and adult musculoskeletal modeling method. Quantifying the strike alteration in the kicking force and muscle force produced by a simple stretching during pregnancy reveals that the stresses and strain stimuli of the fetus show an increasing trend in the second half of pregnancy [33]. At about 35 weeks, the uterine wall is much less deformed than in the first trimester; the fetal bone stresses and strains basically remain unchanged [35]. When the stress and strain stimulation of fetal lower limbs were significantly higher, the intramuscular force, particularly the kicking force, increased. In the third trimester, due to the uterine environment and constrained fetal position, the fetal kicking range decreases while kicking force increases [35]. The growth and development of skeleton is basically driven by cells; through the biomechanical stimulation of fetal tissue, such as stress and strain, the bone gradually mineralizes and forms [74]. With the development of tissue engineering, the existing research is able to simulate the production of engineered skeleton and gristle tissue by studying the natural developmental processes of chondrogenesis and endochondral ossification [75]. The calculation of stress and strain in lower limb bones has also made a great contribution to biomaterials.

### 4.7. Biomechanics of Fetal Lower Limb Movement

Fetal movement can be effectively inferred through monitoring fetal lower limb movement to assess the fetus’ health. Numerous studies have investigated the relationship between fetal lower limb movements and healthy fetus development, as summarized in Table 1. The lower limb movement of the fetus and the whole-body movement of the fetus were firstly distinguished sonographically. The measures included strong movements like fetal trunk movements with kicking and weak movements such as isolated limb motion [28]. The data from 2D ultrasound and 3D ultrasound were used to observe the number of bones and fingers in the lower limb of the fetus through the coronal plane, sagittal plane, and transverse plane, respectively [29]. Abnormal lower limb movements such as limb reduction defects are generally associated with a number of diseases such as skeletal dysplasia or neuromuscular disorders, or neural tube defects. Ultrasound examination of neural tube defects shows the abnormal fetal lower limb starts at 10 weeks onwards, and as a result, isolated lower limb motion occurs [32]. Ultrasound proved to be an effective way to detect deformities and abnormalities in the lower extremities. Compared with MRI detection, real-time ultrasound examination has a certain timeliness because ultrasound can hardly obtain the full body image of the fetus in the third trimester. By comparing different ultrasound views of fetal leg movement in the late trimester, the fetal lower limb movement profile was adequately evaluated with the front of the legs facing the anterior ultrasound probe [32,34]. Combining MRI and ultrasound to confirm the condition of talipes or lower-extremity impairment is highly associated with myelomeningocele or open spinal dysraphism [36]. The Simi Motion System is a software commonly used in sports to measure the motion angles of lower limb joints. Using this software to process ultrasound imaging data, we were able to clearly calculate the change in fetal lower limb movement angle in a pilot experiment [37].

The use of MRI as an adjunct measurement to ultrasound in multiple fetal anomalies has shown beneficial effects, particularly in central nervous disorders. MRI may be valuable for differentiating the etiological heterogeneity that leads to arthrogryposis and fetal akinesia-hypokinesia deformation sequences and identifying related central nervous system abnormalities. Prolonged restriction of fetal movement in the third trimester has a favorable prognosis with the appropriate orthopedic intervention. Conversely, most fetal motor disorders due to congenital neuropathy result in an adverse pregnancy outcome due to hypoplastic lung [30]. Some neurological diseases, such as fetal akinesia deformation sequences, do not respond well to prenatal DNA diagnosis; therefore, prenatal imaging diagnosis plays a vital role in discovering and detecting these diseases [76]. With the improvement in the level of detection accuracy, cine-MRI began to be used to detect the whole fetal movement [69]. On this basis, the examination of fetal lower limbs is becoming increasingly accurate. Developmental hip dysplasia can be analyzed and calculated by cine-MR image sequences to capture the motion at the hip joint [31]. Dysplasia of the hip is the most common abnormal joint shape disease, especially when fetal breech presentation happens with fetal abnormal movement, which then affects prenatal musculoskeletal development and joint shape development [35]. Studies have used adult models and the 2D FE method to calculate fetal lower limb kicking force, and lower limb muscle exertions, stresses and strains [33,35,77]. The average uterus displacements for kicking in utero were calculated using a custom tracking software and the finite element method, then using the musculoskeletal model to predict fetal kicking of the hip joint and knee surrounding maximum muscle forces [33]. By observing the uterine wall deformation and fetal skeleton development, it was found that stress and strain stimulation increases over the second half of pregnancy [77,78]. Altered biomechanical stimulus by stress and strain in the hip joint and kick forces may reveal the link between the risk of developmental dysplasia of the hip and the specific endouterine environment [35]. A series of measurement methods can effectively improve the measurement of fetal lower limb movement.

## 5. Conclusions

After comparing the advantages and disadvantages of different measuring instruments, it is not difficult to conclude that no method is the most ideal for the observation of fetal movement because each method has some limitations. Maternal sensation is the most basic and original method for recording fetal movement, but the data obtained is not reliable due to the intense subjectivity. At present, some maternal wearable devices can capture fetal movements much more accurately than maternal sensation and are not limited by experimental instruments and sites. However, these methods are difficult to distinguish the different fetal movements compared with the imaging information such as ultrasound and MRI. The CTG has been proven not to be a reliable test of fetal movement based on the fetus’ heart rate mode. The actograph is a method that combines the data of CTG and ultrasound. Although ultrasound is the most commonly used fetal monitoring method, it is difficult to thoroughly investigate fetal movement patterns in the third trimester of pregnancy; this problem occurs for the actograph as well. Compared with ultrasound, the cine-MRI may be a better way to quantitatively evaluate fetal movement. However, cine-MRI has its limitations. Because of the limitations and equipment, most pregnant women choose this method only when they feel abnormality in the fetus, so it is difficult to use MRI to obtain more extensive maternal data. Combined with cine-MRI and an adult model to calculate fetal limb movement data, it provides a novel perspective to understand the closed mechanical environment of the uterus. However, adult lower limb biomechanics and in utero fetal movement data still have significant differences, while using the corresponding infant data to simulate the fetal movement may provide more reliable simulation results.

In summary, this review briefly explored the effects of fetus movement on fetal growth and development, the outcomes of fetal movement reduction, as well as methods for measuring fetal movement conditions. This review has a potential role in guiding the treatment and diagnosis of fetal movement reduction and presents a foundation model for the quantification and calculation of fetal movement in the future.

## Figures and Tables

**Table 1:** The detailed measurement of the fetal lower limb movement.

Studies	Gestational Age	Numbers	Reference Method	Ultrasonic Scanning Plane/MRI Section Thickness	Inspection Area
Rayburn et al., 1980 [28]	from 28 to 43 gestational weeks	24 pregnancies	ultrasound	along the longitudinal axis of the fetus	fetal abdomen and lower extremities
Budorick et al., 1998 [29]	from 16 to 32 gestational weeks	33 fetuses	ultrasound	corresponding to the coronal, sagittal, and axial planes	the lower leg bones and digits containing ossification centers; a vertical appearance to hindfoot; visualization of the forefoot, hindfoot, ossification centers in the hindfoot; a perpendicular orientation of the sole of the foot; a complete foot in the axial plane;
Nemec et al., 2011 [30]	from 18 + 4 to 31 + 1 gestational weeks	6 fetuses	MRI	coronal and sagittal T2-w sequences: 3–4 mm; dynamic steady-state free precession sequence: 30 mm; a three-dimensional thick-slab T2-w sequence: 30–50 mm	the whole fetus including the fetal extremities/musculoskeletal system
Giorgi et al., 2015 [31]	three in the early-middle (gestational weeks: 21, 22) stages; two in the late-middle (gestational weeks: 29, 34) stages.	5 fetuses	cine-MRI and Abaqus	30–40 mm	the whole fetus especially the prenatal hip joint
Carreras et al., 2016 [32]	from 18 and 26 gestational weeks	18 fetuses	B-mode ultrasound	sagittal plane	fetal lower limbs movements
Verbruggen et al., 2016 [33]	after 20 gestational weeks	3 fetuses	cine-MRI	30–40 mm	kicking sequences
Maroto et al., 2017 [34]	from 20.6–24.5 gestational weeks	28 fetuses	grey-scale (mode B) ultrasound	a sagittal plane	lower-limb movements
Verbruggen et al., 2018 [35]	20 weeks gestational age	341 fetuses	cine-MRI	30–40 mm	clear in-plane extension-flexion fetal kicks
Oliver et al., 2020 [36]	from 18 to 25 gestational weeks	404 fetuses	ultrasound and MRI	/	fetal lower-extremity
Chen et al., 2021 [37]	24, 27, 30 gestational weeks, respectively	3 fetuses	ultrasound	/	lower-limb movements

## Data Availability

The data that support the findings of this study are available from the corresponding author upon reasonable request.
